# Asymmetric Power Boosts Extortion in an Economic Experiment

**DOI:** 10.1371/journal.pone.0163867

**Published:** 2016-10-04

**Authors:** Christian Hilbe, Kristin Hagel, Manfred Milinski

**Affiliations:** 1 Program for Evolutionary Dynamics, Department of Organismic and Evolutionary Biology and Department of Mathematics, Harvard University, Cambridge MA, United States of America; 2 IST Austria, Klosterneuburg, Austria; 3 Department of Evolutionary Ecology, Max-Planck-Institute for Evolutionary Biology, Plön, Germany; University of Bristol, UNITED KINGDOM

## Abstract

Direct reciprocity is a major mechanism for the evolution of cooperation. Several classical studies have suggested that humans should quickly learn to adopt reciprocal strategies to establish mutual cooperation in repeated interactions. On the other hand, the recently discovered theory of ZD strategies has found that subjects who use extortionate strategies are able to exploit and subdue cooperators. Although such extortioners have been predicted to succeed in any population of adaptive opponents, theoretical follow-up studies questioned whether extortion can evolve in reality. However, most of these studies presumed that individuals have similar strategic possibilities and comparable outside options, whereas asymmetries are ubiquitous in real world applications. Here we show with a model and an economic experiment that extortionate strategies readily emerge once subjects differ in their strategic power. Our experiment combines a repeated social dilemma with asymmetric partner choice. In our main treatment there is one randomly chosen group member who is unilaterally allowed to exchange one of the other group members after every ten rounds of the social dilemma. We find that this asymmetric replacement opportunity generally promotes cooperation, but often the resulting payoff distribution reflects the underlying power structure. Almost half of the subjects in a better strategic position turn into extortioners, who quickly proceed to exploit their peers. By adapting their cooperation probabilities consistent with ZD theory, extortioners force their co-players to cooperate without being similarly cooperative themselves. Comparison to non-extortionate players under the same conditions indicates a substantial net gain to extortion. Our results thus highlight how power asymmetries can endanger mutually beneficial interactions, and transform them into exploitative relationships. In particular, our results indicate that the extortionate strategies predicted from ZD theory could play a more prominent role in our daily interactions than previously thought.

## Introduction

Humans are a remarkably cooperative species [1–6]—we regularly help others, sometimes even if there is no direct or indirect benefit to ourselves [7, 8]. Over the past decades, evolutionary game theory has described various mechanisms that give rise to such cooperative behaviors [9]. For example, models of active partner choice posit that cooperation can thrive if individuals can freely choose with whom to interact, which would lead to a natural bias against defectors [10–14]. As another example, models of direct reciprocity [15, 16] argue that repeated interactions can establish cooperation because our selfish actions today would trigger hostile responses in future.

Many studies in this area have shown how conditionally cooperative strategies can be used to sustain mutual cooperation [16–28]. But the recently discovered class of zero-determinant strategies [29–32] suggests that repeated interactions also allow for more concealed forms of selfishness. By using a reciprocal strategy with cooperation probabilities biased to their own advantage, “extortioners” can lure their co-players into cooperation without being fully cooperative themselves. There is some controversy whether such extortionate practices could evolve in realistic scenarios. Behavioral experiments with human subjects have shown that extortion can be successful in coordination problems in which groups need to contribute towards a well-specified target sum to avoid an economic loss [33]. Also, extortionate strategies perform well if their co-players are told that they would play against an anonymous institution with predefined strategy [34]. But permanent extortion among human subjects over multiple rounds of a social dilemma is more difficult to sustain: in experiments, subjects soon oppose unilateral exploitation [35], and in evolutionary simulations extortioners often switch to more cooperative strategies [36–42].

Most of these previous studies required that the strategic scenario in which the individuals find themselves is symmetric—all subjects have comparable strategic options, and they face similar economic consequences. While such a symmetry assumption is an idealization that often proves useful to derive analytical results, most social interactions involve at least some degree of asymmetry. Once some individuals have more influence than others, individuals in a superior strategic position may well be able to guarantee themselves a higher share of the group payoff, knowing that this comes at the expense of their cooperating peers. Herein, we explore this issue with an economic experiment.

**Fig 1:**
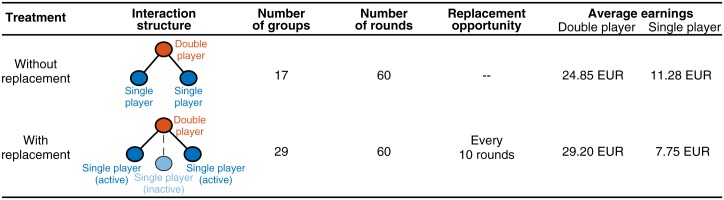
Overview of the experimental setup. Players interact in a repeated prisoner’s dilemma over 60 rounds. In each round they choose between the option C (corresponding to cooperation) and the option D (referring to defection). Payoffs are taken from Axelrod [16]: mutual cooperation yields the reward *R* = € 0.30 for both players, whereas mutual defection gives the punishment payoff *P* = € 0.10; if only one player cooperated, the cooperator receives the sucker’s payoff *S* = € 0.00 and the defector receives the temptation *T* = € 0.50. Both treatments have in common that the game is asymmetric, as double players have twice as many interactions as single players. But the second treatment adds another source of asymmetry, as only double players have the option to replace one of their co-players by the inactive player. The rules of the game, as well as the strategic options of each player were commonly known (except that subjects were not aware of the exact length of the game). In addition, each subject was informed about the decisions of all other group members after each round. For the statistical analysis we considered groups of players as our statistical unit, and we used non-parametric and two-tailed tests throughout. To compare double players with single players, we pooled the decisions of all active single players within a group. Moreover, as double players had more interactions than single players, we considered payoffs per interaction (unless stated otherwise). For the treatment with replacement, the reported total earnings for single players give the average over all three single players (including the inactive player). For details, see Methods.

Our experiment consists of two treatments. In the first treatment we consider a repeated game between two types of players, the so-called double player and two single players. In each round, the double player interacts with each single player in a prisoner’s dilemma. These games are independent: the double player is not required to choose the same action against the two single players, and each single player only interacts with the double player but not with the other single player. The second treatment is similar except that there are three single players, with one of the single players randomly being determined to be initially inactive. Inactive players do not participate in any game, and they do not receive any payoff. After each round, all subjects (including the inactive player) are informed about the decisions of all group members. Every ten rounds, double players can decide whether they want to continue to play with the two currently active single players, or whether they want to choose one of the single players to be replaced by the inactive player (in the latter case, the replaced player becomes the inactive player for the next ten rounds). We refer to these two treatments as the treatment without replacement and the treatment with replacement, respectively (for a summary of the two treatments, see Fig 1). Theory predicts that both treatments have a multitude of possible equilibria, including fair equilibria in which both player types get the same payoff. However, the treatment with replacement may give rise to more extreme outcomes, allowing double players to claim almost all of the generated wealth for themselves (see Methods).

## Results

### Payoffs and cooperation rates across the different treatments

**Fig 2:**
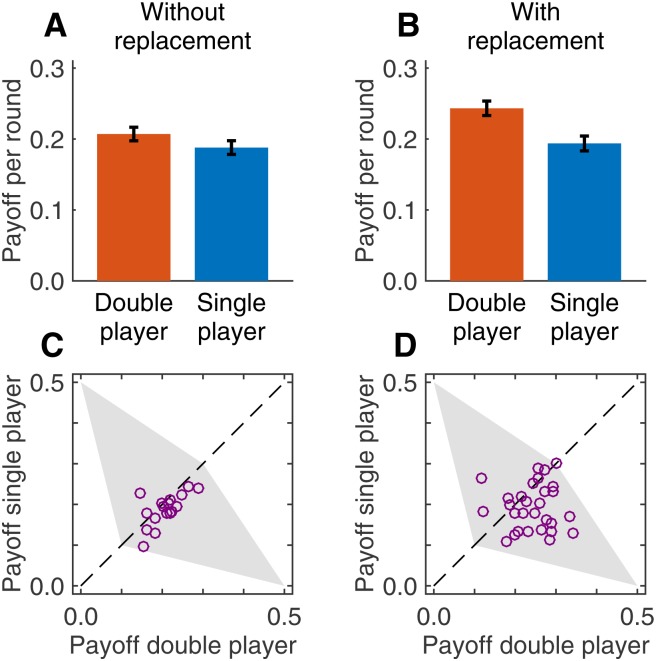
Players earn significantly more when they are allowed to replace group members. The four panels show average payoffs (**A** and **B**) and the overall distribution of payoffs (**C** and **D**) across the two treatments. (**A**) Already without replacement, double players earn more than single players (error bars represent standard errors). (**B**) This payoff advantage becomes even more distinct when the replacement option is available. (**C**,**D**) In the treatment without replacement, groups are clustered around the main diagonal (indicating relatively equal payoffs), whereas in the treatment with replacement, groups are more scattered (indicating considerable payoff differences within some groups).

From our experimental design, it follows that double players had twice as many interactions as single players. Hence, one may expect that they would approximately earn twice as much—but in the experiment they ensured themselves an even bigger share of the cake (Fig 2). Already in the treatment without replacement, double players had slightly higher average payoffs per interaction than single players, presumably because they felt economically more independent (20.7 cents as compared to 18.8 cents, Wilcoxon signed-rank test, *n* = 17, *Z* = 2.464, *p* = 0.014). In the treatment with replacement, the payoff advantage of double players was even more distinct (24.3 cents versus 19.4 cents, Wilcoxon Test, $\hat{n}\;=\;29$, *Z* = 3.018, *p* = 0.003). In particular, there was a treatment effect: double players earned significantly more when they were given the replacement opportunity. In contrast, the payoffs of single players were not significantly affected by the treatment (*p* = 0.829). The lower panels of Fig 2 show the resulting payoff distributions. Without replacement, the two player types typically earned similar payoffs, whereas in the treatment with replacement payoffs are more scattered and biased towards the double player. A superior strategic position thus translated into higher payoffs (see also S1 Fig).

The differences in payoffs were reflected in the subjects’ strategies. In general, all player types exhibited conditionally cooperative behavior, but only in the treatment with replacement we observed a significant increase in cooperation rates over time (S2 Fig). The replacement option raised the single players’ baseline cooperation rate: they had a higher cooperation probability independent of whether or not the double player cooperated in the previous round (S3 Fig). To explore the cause for this increased baseline cooperation rate, we analyzed how double players made their replacement decisions (S4 Fig). Single players were more likely to be replaced when they only cooperated half of the time or less (replacement probabilities are 17.4% and 51.3%, respectively, Wilcoxon test, $\hat{n}=26$, *Z* = 3.749, *p* < 0.001). Similarly, single players were more likely to be replaced when they cooperated less than the other active single player (replacement probabilities increased from 20.6% to 60.9%, Wilcoxon test, $\hat{n}=29$, *Z* = 4.352, *p* < 0.001). These findings allow us to conclude that by using the replacement option, double players had an effective mechanism to enforce cooperation without having to return all acts of cooperation.

### Characterization of extortionate behaviors

The variation between groups in the lower panels in Fig 2B suggests that not all double players made use of their potential to enforce unilateral cooperation. To explore differences in the players’ individual characteristics, we classified double players as extortioners [29] if (i) their cooperation rate was at least 10% lower than the cooperation rate of their single players, and (ii) they incentivized their co-players to cooperate (i.e., the least cooperative single player in a group had a strictly lower payoff than the most cooperative single player—hence it paid for single players to cooperate even when being exploited). In the treatment without replacement, 3 double players satisfied the first criterion, and 13 double players satisfied the second (with 1 out of 17 double players meeting both criteria). In the treatment with replacement, 15 double players satisfied the first criterion, and 22 players satisfied the second (with 13 of 29 double players satisfying both). This comparison shows that the replacement option triggered extortionate behaviors among double players (Fisher’s exact test, *p* = 0.007).

### Performance of extortioners

**Fig 3:**
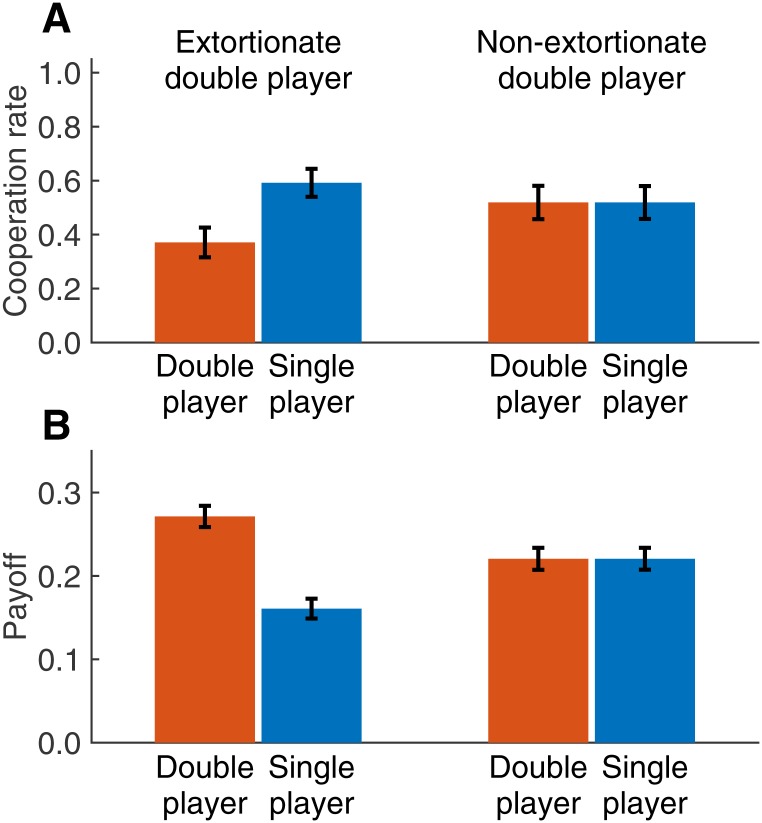
In the treatment with replacement, double players benefit from being extortionate. The graph shows cooperation rates (**A**) and payoffs (**B**) in the treatment with replacement, depending on whether the double player was classified as extortionate or not. Error bars represent standard errors. Extortionate players were less cooperative than non-extortionate players; nevertheless they received more cooperation from their respective co-players. As a result, extortionate double players outperformed both their direct co-players and non-extortionate double players.

Extortionate behaviors paid off, as we can show by comparing the 13 extortioners in the treatment with replacement with the 16 non-extortioners (Fig 3). By definition, extortionate double players were less cooperative than their single players (37.1% versus 59.2%, Wilcoxon test, ${\hat{n}}_{E}\;=\;13$, *Z* = 3.180, *p* = 0.001). In groups without an extortioner, there was no such difference, and both types cooperated in 51.9% of the rounds (see also S5 Fig). Interestingly, although extortioners cooperated less often than non-extortioners, their respective co-players tended to be more cooperative (but not significantly so, Mann-Whitney test, ${\hat{n}}_{E}\;=\;13$, ${\hat{n}}_{N}\;=\;16$, *Z* = 1.009, *p* = 0.329). Overall, double players had a distinct payoff advantage from being extortionate (27.1 cents versus 22.1 cents, Mann-Whitney test, *Z* = 2.500, *p* = 0.012); the difference represents the net benefit to extortion. In contrast, single players had a clear disadvantage from being matched with an extortioner (16.1 cents versus the 22.1 cents they would have received against non-extortioners, Mann-Whitney test, *Z* = 2.851, *p* = 0.004).

**Fig 4:**
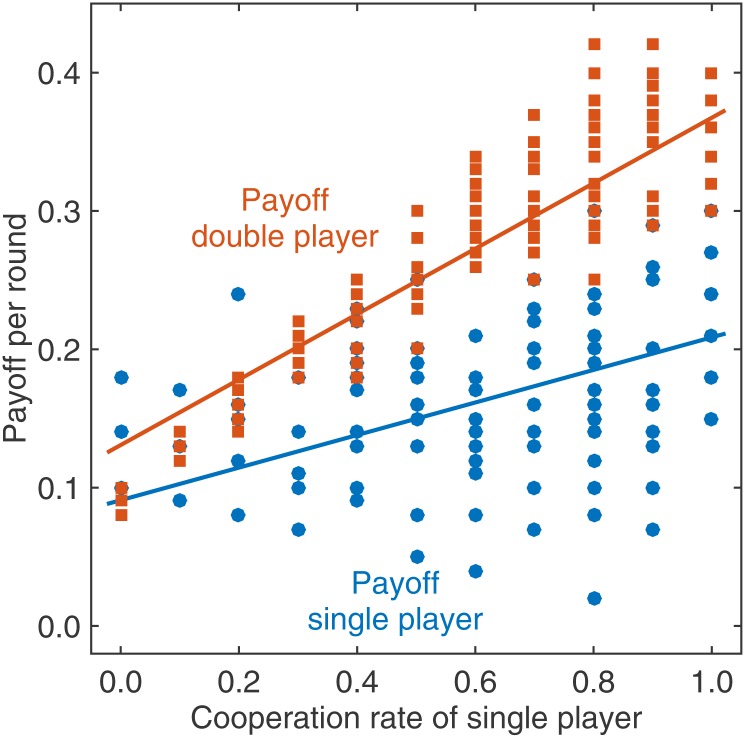
Extortioners incentivize their co-players to cooperate, and they obtain an excessive share of the resulting payoffs. For groups with an extortionate double player in the treatment with replacement, the graph shows how both the single and the double players’ payoffs depend on the single player’s cooperation rate. Each dot corresponds to an outcome of a 10-round block, across the 13 extortionate groups; the two lines represent linear regression curves. Extortioners adopt a strategy such that single players benefit from increasing their cooperation rate within each 10-round block (as the blue line has a positive slope). The more cooperative single players are, the higher is the share of total payoffs that goes to the extortioner (as the distance between the two lines becomes maximal when the single players’ cooperation rate approaches 100%).

Extortion was largely based on exploitation (56.5% of the payoffs of the double player stemmed from rounds in which the double player chose D and earned € 0.50 while the single player chose C and earned € 0.00). To enforce such a beneficial payoff relationship, extortionate players were more selective concerning their co-players: those single players who were least cooperative had a lower number of active rounds (30 rounds compared to 35.3 rounds in groups with a non-extortioner, Mann-Whitney test, *Z* = 2.443, *p* = 0.020). Consequently, the least cooperative player also had a lower total payoff when interacting with an extortioner (in total they earned 4.59 Euros when interacting with an extortioner, and 7.53 Euros against a non-extortioner, Mann-Whitney test, *Z* = 2.983, *p* = 0.002). But even within each 10-round block, extortioners strongly incentivized their opponents to cooperate—but in a way that secured that they would always get a larger portion of any surplus. Against an extortioner, single players could only gain by increasing their own cooperation rate, from which the extortioner would gain even more, as correctly predicted by ZD theory [29] (Fig 4; for a statistical analysis using a generalized linear model, see S1 Table and S6 Fig).

## Discussion

In the last few years, several theoretical studies have explored which kind of repeated interactions allow for extortion [29, 43–46], and which evolutionary scenarios actually favor its emergence [36–41, 47]. While this line of work has shown that extortionate strategies exist in almost any natural setup, they almost never evolve. Recent experimental evidence points to a similar direction. Extortion may evolve in simple coordination problems where players risk losing their whole endowment if groups fail to reach a well-specified target sum [33], but in repeated prisoner’s dilemmas, humans often resist such forms of exploitation. They are willing to engage in costly episodes of mutual defection to withdraw the extortioner’s excessive gain [35], especially if the game is only repeated for relatively few rounds [34].

Herein we have reported results from a behavioral experiment showing that extortion readily emerges when players differ in their strategic power. Our design considers groups of players who engage in pairwise prisoner’s dilemma games; but only one of the group members, the so-called double player, is allowed to replace uncooperative co-players. This asymmetric replacement option generally increased overall cooperation rates—just as predicted by traditional models of partner choice [10–14]. However, the resulting wealth was shared unevenly—even after accounting for the fact that double players had more interactions than their co-players, double players earned 25% more. The asymmetric replacement option thus allowed double players to sustain a prolonged exploitative relationship. However, it is also important to note that only 45% of the double players actually made use of this opportunity—a slight majority of double players refused to take unilateral advantage of their peers.

The extortioners who performed best in our experiment did not act as mere defectors. Like Tit-for-Tat players, they fully reciprocate defection—but unlike Tit-for-Tat, they reciprocate cooperation only partially (in 42.9% of the cases, compared to the single players’ cooperation probability of 78.0%, S3 Fig). Against an extortioner, the best the single player can do is to give in and to cooperate, even if this means that the extortioner receives a disproportionate share of the resulting surplus [29]. Such extortioners may be identified with Machiavellian-type leaders, who consistently use coercion and persuasion to gain permanent advantages [48, 49]. In post-experiment questionnaires, the vast majority of double players expressed they were aware of their superior strategic position, whereas single players knew that they needed to appease their co-player. Only a few single players tried to oppose an extortionate double player, and those few players were typically displaced soon. The questionnaires suggest that by the end of the experiment, many of the replaced subjects regretted their early actions, but then it was too late. Under such conditions, power asymmetries can easily pervert mutually beneficial interactions, and thereby favor the emergence of extortionate relationships.

The existence of such extortionate strategies has been discovered only recently [29], but only few empirical tests exist. Herein, we have shown that asymmetry can open the door for these new ZD extortion strategies. The specific design of our experiment seems to feature certain aspects of labour markets, but we believe that extortionate practices can emerge in many other asymmetric social interactions too; they only need to be uncovered.

## Materials and Methods

### Experimental methods

Experiments were conducted in May and November 2014 with 167 first-year biology students at the universities of Kiel and Hamburg, Germany. We invited 6–8 volunteers to each experimental session (by running two experimental games per session in parallel we aimed to create a more anonymous environment). Before each session, subjects were orally informed by one of the experimenters (M. M.) about how to operate the computers, and about the measures that were taken to ensure the subjects’ anonymity. During the experiment, subjects had a pseudonym, were separated by opaque partitions, and they were instructed not to talk to each other during or after the experiment. Subjects made all their decisions through a computer interface based on z-Tree [50]. Each desk was equipped with paper and pencil, and subjects were encouraged to take notes to make informed decisions. Average earnings for the experiment are reported in Fig 1. Subjects knew they would receive their earnings anonymously and in cash directly after the game. In addition, each participant received a €10 show-up fee. Including the initial instruction phase, the experiment took ∼ 1.5 hours.

The first treatment (“without replacement”) consisted of a repeated prisoner’s dilemma game between two types of players, a “double player” and two “single players”. In each round, the double player interacted with each of the two single players, whereas the two single players only interacted with the double player, but not with each other. The double player was not required to choose the same action against the two single players. The second treatment (“with replacement”) was similar except that there were three single players, with one of the single players initially being randomly determined to be inactive. The inactive player did not participate in any game and did not receive any payoff. Every ten rounds the double player could decide whether he wanted to continue to play with the two currently active single players, or whether he wanted to replace one of them by the currently inactive player. In the latter case, the replaced single player became inactive for the next 10 rounds (at which point the double player could again decide with whom to continue). The game consisted of 60 rounds (subjects were not informed about the exact duration of the game, but rather that they would play over many rounds). For a detailed explanation of each treatment, we provide a translation of the instructions in the Supplementary Information.

Participation in this economic experiment was voluntary. All participants were university students and have reached the age of majority. The experiment did not involve any particular foreseeable risks or discomforts for the participants. All decisions during the experiment were made anonymously, and no personal information (e.g., gender, age, etc.) was recorded. We did not seek approval from an ethics committee, since German regulations do not require such an approval for behavioral experiments in which individuals are anonymous, and in which individuals do not face any particular risks for their physical or psychological well-being. We have received the subjects’ verbal consent to participate, and subjects had the option to stop participating at any time (but nobody did). For behavioral experiments as ours, written consent to participate is not required according to German regulations.

### Theoretical predictions

Unlike other experimental games with asymmetries, such as the ultimatum game [51], the game used herein allows us to order players with respect to their strategic options. With replacement, double players have strictly more strategic power than single players, which should have consequences on the possible equilibrium outcomes. Assuming infinitely many rounds and no discounting, we can use the Folk theorem of repeated games to gain some basic insights. According to the Folk theorem, all feasible payoff pairs above the minmax payoffs can be sustained in equilibrium [52]. In the treatment without replacement, players can guarantee themselves the mutual defection payoff of € 0.10 per round. Thus we would expect that the realized payoffs (*π*_*D*_, *π*_*S*_) of double players and single players are within the set

$$E=\left\{(\pi_{D},\pi_{S})\in F\;|\;\pi_{D}\geq 0.1,\;\pi_{S}\geq 0.1\;\right\},$$ (1)

where $F\in R^{2}$ is the convex hull of all one-shot payoffs (i.e., the grey-shaded area in Fig 2). In contrast, in the treatment with replacement single players can become inactive, and thus each single player’s minmax payoff is zero. There are equilibria in which none of the active single players is replaced, and the average payoffs per interaction of the double player and of each active single players is in the set

$$\hat{E}=\left\{(\pi_{D},\pi_{S})\in F\;|\;\pi_{D}\geq 0.1,\;\pi_{S}\geq 0\right\},$$ (2)

with the payoff of the inactive player being zero. Comparing E with $\hat{E}$ shows that both treatments allow for “fair” equilibria, in which the double player gets the same payoff per interaction as each (active) single player. But in the treatment with replacement, the double player has additional options: the replacement option allows for equilibria in which the double player defects in almost every round along the equilibrium path, whereas both active single players are fully cooperative.

## Supporting Information

S1 FigIn the treatment with replacement, double players were more likely to receive high payoffs.The figure shows the distribution of payoffs per interaction for the treatment without replacement (**A** and **B**) and for the treatment with replacement (**C** and **D**). Comparing the payoffs of double players, the graph illustrates that the payoff distribution is shifted to the right in the treatment with replacement (Mann-Whitney Test, *n* = 17, $\hat{n}=29$, *Z* = 2.424, *p* = 0.015). Only with that replacement option, a majority of double players earned more than 25 Cents per interaction. In particular, only in the treatment with replacement there were instances of double players earning more than the mutual cooperation payoff *R* = 30 Cents (the maximum payoff obtained by a double player in our experiment was 34.3 Cents per interaction). In contrast, comparing the payoffs of single players did not reveal any significant differences (Mann-Whitney Test, *n* = 17, $\hat{n}=29$, *Z* = 0.216, *p* = 0.829).(EPS)

S2 FigThe replacement option leads to a significant trend towards more cooperation.Overall, cooperation rates in the treatment without replacement were 38.6% (double players) and 42.4% (single players), whereas cooperation rates in the treatment with replacement were 45.3% (double players) and 55.2% (single players). The two panels illustrate the dynamics of cooperation over the course of the game. (**A**) In the treatment without replacement, cooperation rates increased, but not significantly. Double players raised their cooperation rates from 31.5% during the first 20 rounds to 45.6% during the last 20 rounds (Wilcoxon test, *p* = 0.201), whereas the cooperation rates of single players increased from 35.0% to 49.6% (Wilcoxon test, *p* = 0.190). (**B**) With replacement, both types of players became significantly more cooperative over time: double players increased their cooperation rate from 37.1% to 53.9% (Wilcoxon test, $\hat{n}=29$, *Z* = 3.307, *p* = 0.001), and single players increased their cooperation rates from 43.5% to 64.4% (Wilcoxon test, *Z* = 3.793, *p*<0.001). We note that the increase in cooperation rates of single players in the treatment with replacement could be merely due to the fact that double players were able to replace non-cooperative single players by more cooperative inactive players (without having an effect on individual cooperation rates). But even when we considered each single player individually, we still found a significant increase (from 43.6% during the single player’s first 10 active rounds to 56.9% for the 10 last active rounds, Wilcoxon test, *Z* = 2.705, *p* = 0.007).(EPS)

S3 FigSingle players in the treatment with replacement showed an increased baseline cooperation probability.The graphs depict the players’ cooperation probabilities, depending on the co-player’s decision in the previous round (error bars represent standard errors). Subjects were generally conditionally cooperative: they were more likely to cooperate if their co-player cooperated in the previous round. (**A**) In the treatment without replacement, a double player’s cooperation probability after C was 59.0%, whereas the double player’s probability to cooperate after D was only 18.1% (Wilcoxon test, *n* = 17, *Z* = 3.575, *p*<0.001). The numbers for single players were similar: their cooperation probability after C was 55.8%, and the cooperation probability after D was 31.2% (Wilcoxon test, *Z* = 2.959, *p* = 0.003). (**B**) In the treatment with replacement, double players cooperated in 55.4% of the cases in which the co-player played C in the previous round, whereas the cooperation rate dropped to 25.0% if the co-player played D (Wilcoxon test, $\hat{n}\;=\;29$, *Z* = 2.903, *p*<0.001). For single players the respective numbers are 68.4% after C, and 40.2% after D (Wilcoxon test, *Z* = 4.315, *p*<0.001). In particular, independent of whether the co-player chose C or D in the previous round, single players had a higher cooperation probability than double players (after C: Wilcoxon test, *Z* = 2.220, *p* = 0.026; after D: Wilcoxon test, *Z* = 2.811, *p* = 0.005). (**C**) For groups with an extortioner, the strategies of double players and single players were even more distinct. Double players were less likely to cooperate after C (42.9% as compared to 78.0% for single players), and they were also less likely to cooperate after D (20.5% as compared to 43.9%).(EPS)

S4 FigSingle players were most likely to be replaced if they either cooperated less than the other active single player, or in less than half of the rounds.In this graph, the *x*-axis gives the cooperation rate of a focal single player, and the *y*-axis shows the cooperation rate of the other active single player during a 10-round block. A square is used to show that the respective case was observed (the respective number within the square shows how often this case was observed). To display whether the focal single player was replaced we use purple (corresponding to a replacement probability of 100%) or orange (replacement probability of 0%). Purple squares are predominantly in the upper left corner (i.e., above the diagonal), showing that single players were most likely to be replaced if they cooperated less often than the other single player. When we restrict attention to cases when both single players cooperated in more than half of the rounds, it was still the single player who cooperated less who was more likely to be replaced (12.0% as compared to 41.7%, Wilcoxon test, $\hat{n}=15$, *Z* = 2.366, *p* = 0.018). Conversely, if we only consider single players who cooperated less than the other single player, the chance to become replaced was higher when the single player cooperated only in half of the rounds or less (41.7% versus 66.3%, Wilcoxon test, $\hat{n}=14$, *Z* = −2.289, *p* = 0.022).(EPS)

S5 FigExtortionate players decrease their cooperation rate towards the end of each 10-round block.The curves show how often players cooperate in a given round of a 10-round block, aggregated over all 6 blocks of a game (error bars represent standard errors). (**A**) Extortionate double players had a lower cooperation rate than their co-players. This cooperation gap becomes particularly clear in Periods 7–10, since extortioners became significantly less cooperative by the end of each block (in the first 5 rounds of each block their cooperation probability was 43.8%, whereas in the last 5 rounds their cooperation rate dropped to 30.4%. Wilcoxon test, ${\hat{n}}_{E}=13$, *Z* = 2.595, *p* = 0.009). (**B**) Groups with non-extortionate double players did not exhibit a similar trend within the 10-round blocks (the cooperation rates of double players were 53.6% and 50.1%, respectively. Wilcoxon test, ${\hat{n}}_{N}=16$, *Z* = 1.164, *p* = 0.244). Similarly, there was no significant drop in cooperation rates within the 10-round blocks among the single players (neither for extortionate groups nor for non-extortionate groups).(EPS)

S6 Fig Relationship between the cooperation rates of single players and payoffs. This figure extends Fig 4 from the main text; each data point corresponds to an interaction between a single player and the double player during a 10-round block. Blue circles show the payoff of the single player, red squares give the payoff of the double player. Blue and red lines show the linear trend according to a least square regression. We consider three classes of groups: (**A**) groups in the treatment without replacement; (**B**) groups in the treatment with replacement, and in which the double player was classified as extortioner, and (**C**) groups in the treatment with replacement without extortioner. In all three classes, the single player’s cooperation rate had a strongly positive effect on the double player’s payoff, and a weakly positive effect on the single player’s payoff. But only against extortioners, the red curve is strictly above the blue curve.
(EPS)

S1 Table A generalized linear model confirms that the single player’s cooperation rate is positively correlated with the single player’s own payoff, and with the double player’s payoff. This table complements S6 Fig; it presents results from a generalized linear model in which we explore how the players’ payoffs depend on (i) the cooperation rate of the single player, (ii) whether the group contains an extortionate double player (only for the treatment with replacement), and (iii) on a possible interaction between the type of the double player and the single player’s cooperation rate. The cooperation rate of the single player has a positive effect on all considered quantities. In addition, the extortion variable has a negative effect on the single players’ payoff and a positive effect on the double player’s payoff. Our model is based on the data of 10-round blocks; to account for the fact that different blocks of the same group cannot be considered as independent, the error terms are clustered by group.
(PDF)

S1 File Experimental game instructions. In the beginning of our experiment, subjects were asked to read a few pages on their computer screens that would explain the rules of the subsequent game. Here, we provide these instructions, translated from German.
(PDF)

S2 File Experimental Data. The raw data file containing the experimental decisions of all our study subjects.
(XLSX)
