# Supplementary material for: Asymmetric Power Boosts Extortion in an Economic Experiment
Source: PLoS One. 2016 Oct 4;11(10):e0163867. doi: 10.1371/journal.pone.0163867 (PMC5049762; doi:10.1371/journal.pone.0163867)
Supplement: S1 Table — This table complements S6 Fig; it presents results from a generalized linear model in which we explore how the players’ payoffs depend on (i) the cooperation rate of the single player, (ii) whether the group contains an extortionate double player (only for the treatment with replacement), and (iii) on a possible interaction between the type of the double player and the single player’s cooperation rate. The cooperation rate of the single player has a positive effect on all considered quantities. In addition, the extortion variable has a negative effect on the single players’ payoff and a positive effect on the double player’s payoff. Our model is based on the data of 10-round blocks; to account for the fact that different blocks of the same group cannot be considered as independent, the error terms are clustered by group. (PDF) [file pone.0163867.s007.pdf]

	Dependent variable: Payoff single player, π^S		Dependent variable: Payoff double player, π^D		Dependent variable: Payoff difference, $\pi^D - \pi^S$	
	**Without replacement**	**With replacement**	**Without replacement**	**With replacement**	**Without replacement**	**With replacement**
Constant	0.1270^{***}	0.1775^{***}	0.1135^{***}	0.0963^{***}	-0.0142	-0.0813^{***}
	(0.0086)	(0.0120)	(0.0034)	(0.0071)	(0.0118)	(0.0187)
Cooperation rate of single player	0.1438^{***}	0.0830^{***}	0.2207^{***}	0.2395^{***}	0.0787^{***}	0.1567^{***}
	(0.0142)	(0.0141)	(0.0066)	(0.0077)	(0.0204)	(0.0212)
Dummy Extortion		-0.0746^{***}		0.0244^{*}		0.0990^{**}
		(0.0185)		(0.0108)		(0.0287)
Extortion *Cooperation rate		0.0147		0.0148		0.0001
		(0.0210)		(0.0114)		(0.0316)

S1 Table. A generalised linear model confirms that the single player's cooperation rate is positively correlated with the single player's own payoff, and with the double player's payoff. This table complements S6 Fig; it presents results from a generalised linear model in which we explore how the players' payoffs depend on (i) the cooperation rate of the single player, (ii) whether the group contains an extortionate double player (only for the treatment with replacement), and (iii) on a possible interaction between the type of the double player and the single player's cooperation rate. The cooperation rate of the single player has a positive effect on all considered quantities. In addition, the extortion variable has a negative effect on the single players' payoff and a positive effect on the double player's payoff. Our model is based on the data of 10-round blocks; to account for the fact that different blocks of the same group cannot be considered as independent, the error terms are clustered by group.
