# Supplementary material for: Asymmetric Power Boosts Extortion in an Economic Experiment
Source: PLoS One. 2016 Oct 4;11(10):e0163867. doi: 10.1371/journal.pone.0163867 (PMC5049762; doi:10.1371/journal.pone.0163867)
Supplement: S1 File — In the beginning of our experiment, subjects were asked to read a few pages on their computer screens that would explain the rules of the subsequent game. Here, we provide these instructions, translated from German. (PDF) [file pone.0163867.s008.pdf]

Supplementary methods: Experimental game instructions.

In the beginning of our experiment, subjects were asked to read a few pages on their computer screens that would explain the rules of the subsequent game. In the following, we provide these instructions, translated from German.

Instructions for the treatment without replacement:

Page 1. Welcome to this experiment in which you can earn money. In the beginning of this experiment, 10 Euros will be credited to your account. During the experiment you can earn additional money. The exact amount of money that you earn will depend on your own decisions and on the decisions of the other participants. All decisions you make are anonymous. To ensure this, the computer program assigns you a pseudonym, which you can see at the bottom left of your screen. These pseudonyms correspond to names of moons in our solar system. At the end of the game, you will receive the money that you have earned in cash anonymously under your pseudonym. To render this experiment successful, it is strictly forbidden for participants to talk to each other or to communicate in any other way. After having read this text completely, please confirm by pressing the 'Ok'-button.

Page 2. The following introduction pages will explain the rules of the experiment. The introduction is the same for all participants. During the experiment, you will play in a group with two other participants, which the computer assigns randomly to your group. The composition of your group will remain the same over the entire game. Within the group, there are two categories of players: a double player and two single players. Your category is chosen randomly in the beginning of the experiment. All players keep their category over the whole course of the game. After having read this text completely, please confirm by pressing the 'Ok'-button.

Page 3. During the experiment, you are playing several rounds of a simple decision situation. In these decision situations the double players interacts independently with each of the single players. In each decision situation, two players need to simultaneously choose a letter (either **C** or **D**). Each player needs to decide without knowing the choice of the co-player. Depending on your decision and on the decision of your co-player, you receive a certain payoff. The following table shows the possible payoffs. The first amount in each cell corresponds to your own payoff, and the second amount corresponds to the payoff of your co-player.

		Decision of your co-player: **C**	Decision of your co-player: **D**
Your decision	**C**	€ 0.30, € 0.30	€ 0.00, € 0.50
	**D**	€ 0.50, € 0.00	€ 0.10, € 0.10

Thus, there are four possible outcomes:

You: **C**	Your co-player: **C**	You get € 0.30	Your co-player gets € 0.30
You: **C**	Your co-player: **D**	You get € 0.00	Your co-player gets € 0.50
You: **D**	Your co-player: **C**	You get € 0.50	Your co-player gets € 0.00
You: **D**	Your co-player: **D**	You get € 0.10	Your co-player gets € 0.10

The double player makes two decisions in each round (one decision for each of the two single players). On the other hand, single players only make one decision (single players only play with the double player but not with each other). You don't need to remember the above table – you will be shown the table in each round again. After having read this text completely, please confirm by pressing the 'Ok'-button.

Page 4. **Examples:** Consider a group with three participants named Portia, Carpo and Galatea. The computer determines randomly that Portia is chosen as the double player, and that Carpo and Galatea are the two single players. Portia is asked in every round:

Which letter do you choose against Carpo (C or D)?
Which letter do you choose against Galatea (C or D)?

Portia is allowed to answer both questions independently. The two single players, Carpo and Galatea, are only shown one question:

Which letter do you choose against Portia (C or D)?

After all participants have made their decisions, all participants are shown the result of this round.

Example 1:

Portia has played C against Carpo and gets € 0.30.
Carpo has played C against Portia and gets € 0.30.

Portia has played D against Galatea and gets € 0.50.
Galatea has played C against Portia and gets € 0.00.

Example 2:

Portia has played C against Carpo and gets € 0.00.
Carpo has played D against Portia and gets € 0.50.

Portia has played D against Galatea and gets € 0.10.
Galatea has played D against Portia and gets € 0.10.

Once all players have confirmed this outcome summary by clicking on the 'Ok' button, this round is over. All players get their respective amount credited to their account (in the first example Carpo earns € 0.30, Galatea earns € 0.00, and Portia earns € 0.30 + € 0.50 = € 0.80). The experiment consists of many rounds. In each round you are exposed to the same decision situation (however, you are free to make a different decision in each round). After having read this text completely, please confirm by pressing the 'Ok'-button.

Page 5. The experiment starts now! You have a credit of 10 Euros on your account. After having read this text completely, please confirm by pressing the 'Ok'-button.

Instructions for the treatment with replacement:

Page 1. Welcome to this experiment in which you can earn money. In the beginning of this experiment, 10 Euros will be credited to your account. During the experiment you can earn additional money. The exact amount of money that you earn will depend on your own decisions and on the decisions of the other participants. All decisions you make are anonymous. To ensure this, the computer program assigns you a pseudonym, which you can see at the bottom left of your screen. These pseudonyms correspond to names of moons in our solar system. At the end of the game, you will receive the money that you have earned in cash anonymously under your pseudonym. To render this experiment successful, it is strictly forbidden for participants to talk to each other or to communicate in any other way. After having read this text completely, please confirm by pressing the 'Ok'-button.

Page 2. The following introduction pages will explain the rules of the experiment. The introduction is the same for all participants. During the experiment, you will play in a group with two other participants, which the computer assigns randomly to your group. The composition of your group will remain the same over the entire game. Within the group, there are three categories of players: a double player, two active single players, and an inactive single player. Your category is chosen randomly in the beginning of the experiment. Double players keep their category over the whole course of the game. But over the course of the experiment, active single players can become inactive single players (and vice versa). After having read this text completely, please confirm by pressing the 'Ok'-button.

Page 3. During the experiment, you are playing several rounds of a simple decision situation. In these decision situations the double players interacts independently with each of the active single players. In each decision situation, two players need to simultaneously choose a letter (either **C** or **D**). Each player needs to decide without knowing the choice of the co-player. Depending on your decision and on the decision of your co-player, you receive a certain payoff. The following table shows the possible payoffs. The first amount in each cell corresponds to your own payoff, and the second amount corresponds to the payoff of your co-player.

		Decision of your co-player: **C**	Decision of your co-player: **D**
Your decision	**C**	€ 0.30, € 0.30	€ 0.00, € 0.50
	**D**	€ 0.50, € 0.00	€ 0.10, € 0.10

Thus, there are four possible outcomes:

You: **C**	Your co-player: **C**	You get € 0.30	Your co-player gets € 0.30
You: **C**	Your co-player: **D**	You get € 0.00	Your co-player gets € 0.50
You: **D**	Your co-player: **C**	You get € 0.50	Your co-player gets € 0.00
You: **D**	Your co-player: **D**	You get € 0.10	Your co-player gets € 0.10

The double player makes two decisions in each round (one decision for each of the two active single players). On the other hand, active single players only make one decision (active single players only play with the double player but not with each other). The inactive single player is not able to make a decision in that round (as a consequence, the inactive single player also has no opportunity to earn money in that round). You don't need to remember the above table – you will be shown the table in each round again. After having read this text completely, please confirm by pressing the 'Ok'-button.

Page 4. **Examples:** Consider a group with four participants named Portia, Carpo, Galatea and Triton. The computer determines randomly that Portia is chosen as the double player, Carpo and Galatea are the two active single players, and Triton is the inactive single player. Portia is asked in every round:

Which letter do you choose against Carpo (C or D)?
Which letter do you choose against Galatea (C or D)?

Portia is allowed to answer both questions independently. The two single players, Carpo and Galatea, are only shown one question:

Which letter do you choose against Portia (C or D)?

The inactive single player Triton cannot make a decision in this round. After all participants have made their decisions, all participants (including the inactive single player) are shown the result of this round.

Example 1:
Portia has played C against Carpo and gets € 0.30.
Carpo has played C against Portia and gets € 0.30.

Portia has played D against Galatea and gets € 0.50.
Galatea has played C against Portia and gets € 0.00.

Example 2:
Portia has played C against Carpo and gets € 0.00.
Carpo has played D against Portia and gets € 0.50.

Portia has played D against Galatea and gets € 0.10.
Galatea has played D against Portia and gets € 0.10.

Once all players have confirmed this outcome summary by clicking on the 'Ok' button, this round is over. All players get their respective amount credited to their account (in the first example Carpo earns € 0.30, Galatea earns € 0.00, Portia earns € 0.30 + € 0.50 = € 0.80, and the inactive single player Triton automatically gets € 0.00). The experiment consists of many rounds. In each round you are exposed to the same decision situation (however, you are free to make a different decision in each round). After having read this text completely, please confirm by pressing the 'Ok'-button.

Page 5. In addition to the above decision situation, the double player has the option to replace one of the active single players by the inactive single player every ten rounds.

Example: As before, suppose the computer has determined Portia to be the double

player, Carpo and Galatea are the active single players, and Triton is the inactive single player. After 10 rounds Portia is asked:

You have the option to choose one of your recent co-players (Carpo and Galatea) to be replaced by Triton. What is your decision?

Portia can choose among three possible answers:

Keep Carpo and Galatea
Replace Carpo by Triton
Replace Galatea by Triton

During that time, the other group members cannot make any decision. Once Portia has made a decision, the result is shown to all group members. For example:

Portia has decided to replace Galatea, and to continue playing with Carpo and Triton.

In that case, Galatea becomes the inactive single player, and for the next ten rounds Carpo and Triton are the active single players. Every ten rounds, Portia can again decide whether to replace one of the active single players. After having read this text completely, please confirm by pressing the 'Ok'-button.

Page 6. The experiment starts now! You have a credit of 10 Euros on your account. After having read this text completely, please confirm by pressing the 'Ok'-button.
